# Deregulation of protein phosphatase 2A inhibitor SET is associated with malignant progression in breast cancer

**DOI:** 10.1038/s41598-021-93620-y

**Published:** 2021-07-09

**Authors:** Katsunori Tozuka, Pattama Wongsirisin, Shigenori E. Nagai, Yasuhito Kobayashi, Miki Kanno, Kazuyuki Kubo, Ken Takai, Kenichi Inoue, Hiroshi Matsumoto, Yoshihito Shimizu, Masami Suganuma

**Affiliations:** 1 Division of Breast Surgery, Saitama Cancer Center, Saitama, Japan; 2 Graduate School of Science and Engineering, Saitama University, Saitama, Japan; 3 Research Institute for Clinical Oncology, Saitama Cancer Center, Saitama, Japan; 4 Division of Breast Oncology, Saitama Cancer Center, Saitama, Japan; 5 Saitama Cardiovascular and Respiratory Center, Saitama, Japan

**Keywords:** Cancer, Cell biology

## Abstract

To understand the mechanism underlying metastasis, identification of a mechanism-based and common biomarker for circulating tumour cells (CTCs) in heterogenous breast cancer is needed. SET, an endogenous inhibitor of protein phosphatase 2A, was overexpressed in all subtypes of invasive breast carcinoma tissues. Treatment with SET-targeted siRNAs reduced the motility of MCF-7 and MDA-MB-231 cells in transwell assay. SET knockdown reduced the number of mammospheres by 60–70% in MCF-7 and MDA-MB-231 cells, which was associated with the downregulation of *OCT4* and *SLUG*. Hence, we analysed the presence of SET-expressing CTCs (SET-CTCs) in 24 breast cancer patients. CTCs were enriched using a size-based method and then immunocytochemically analysed using an anti-SET antibody. SET-CTCs were detected in 6/6 (100%) patients with recurrent breast cancer with a median value of 12 (12 cells/3 mL blood), and in 13/18 (72.2%) patients with stage I–III breast cancer with a median value of 2.5, while the median value of healthy controls was 0. Importantly, high numbers of SET-CTCs were correlated with lymph node metastasis in patients with stage I–III disease. Our results indicate that SET contributes to breast cancer progression and can act as a potential biomarker of CTCs for the detection of metastasis.

## Introduction

Breast cancer is a heterogeneous disease classified into four clinical subtypes based on the expression of hormone receptors (HR), including oestrogen and progesterone receptors (ER/PR) and human epidermal growth factor receptor 2 (HER2)^1^. Although adjuvant systemic therapy for breast cancer patients improves survival, some patients still eventually develop metastatic lesions. Early detection of metastasis, as well as the development of new mechanism-based agents, is important for overcoming metastatic disease. To achieve this goal, a comprehensive understanding of the characteristics of primary tumours and cancer cells in circulation, called circulating tumour cells (CTCs), is critical, as CTCs are believed to be “seeds” of metastasis^2,3^. Currently, the enumeration of epithelial cell adhesion molecule (EpCAM)-positive CTCs (EpCAM-CTCs) has allowed estimation of overall metastatic burden in patients with breast cancer^4^. However, EpCAM-CTCs are undetectable in one third of patients with metastatic disease^5^. As many cancer cells lose epithelial features due to epithelial-mesenchymal transition (EMT) during metastasis^6,7^, identification of a new molecule that plays a key role in metastasis and that is expressed in CTCs is necessary. The molecule is expected to help improve early detection of metastasis and evaluation of treatment response in patients.

SET is a 39-kDa oncoprotein encoded by the *set* gene, which was discovered as a component of a *set-can* fusion gene in acute myeloid leukemia (AML)^8^. Subsequently, Li et al. observed that SET protein is a potent and specific inhibitor of protein phosphatase 2A (PP2A) based on the results that a heat-stable PP2A inhibitor called I_2_^PP2A^ was a truncated form of SET^9,10^. PP2A is a major serine/threonine phosphatase regulating multiple signalling pathways, including those involved in cell proliferation and metastasis, which antagonises kinase activities^11^. The inhibition of PP1 and PP2A is a common mechanism underlying tumour promotion in various organs of rodents: The okadaic acid class compounds, including okadaic acid, dinophysistoxin-1, calyculin A, microcystin-LR, and nodularin, are chemical inhibitors of PP1 and PP2A and induce tumour-promoting activities in mouse skin, rat glandular stomach, and rat liver^12^. Furthermore, the transforming simian vacuolating virus 40 (SV40) small T-antigen inhibits PP2A activity and transforms human cells^13^. Unusual inhibition of PP2A promotes the malignant transformation of normal cells. Studies have shown that PP2A is a unique tumour suppressor, which is rarely mutated or deleted, but its function is impaired by “inhibitor proteins”^14^. Abnormal overexpression of cellular PP2A inhibitors induces dysregulation of signalling pathways by increasing the levels of phosphoproteins, similar to abnormal activation of serine/threonine kinases. SET is a physiological endogenous PP2A inhibitor that is 30-fold weaker than the okadaic acid class of compounds^12^. Importantly, SET protein levels are high in various cancers, including breast, colon, pancreas, gastric, liver and lung cancers. Likewise, SET protein levels were also found to be high in leukemia, including chronic myeloid leukemia (CML) and acute myeloid leukemia (AML) and lymphoma^15–22^. Overexpression of SET perturbs various signalling pathways, and high SET levels correlate with drug resistance and a poor prognosis in various human cancers. In addition to SET, cancerous inhibitor of PP2A (CIP2A), another well-known cellular inhibitor of PP2A, is often overexpressed in various cancer tissues^23,24^. Among the known cellular inhibitors of PP2A (SET, CIP2A and protein phosphatase methylesterase 1 (PME-1)), SET is the most potent and regulates 30.5% of the phosphopeptides dephosphorylated by PP2A. In addition, the majority of the CIP2A- and PME-1-target peptides overlapped with the target peptides of SET^25^. Furthermore, targeting of SET with siRNA also down-regulates CIP2A^26^. Therefore, we hypothesised that SET might be the desired target molecule, if it is expressed in CTCs.

In this study, we found that SET was overexpressed in breast cancer tissues of all subtypes. Targeting SET using siRNA in two breast cancer cell lines, MCF-7 (HR-positive) and MDA-MB-231 (triple-negative), revealed that SET plays a significant role in controlling motility and stemness. Using a size-based enumeration method, we identified heterogenous CTCs in breast cancer patients, as reported previously^27,28^. We compared numbers of SET-expressing CTCs (SET-CTCs) with those of the epithelial-CTCs (expressing pan-cytokeratin and EpCAM) and mesenchymal-CTCs (expressing vimentin and gross cystic disease fluid protein-15 (GCDFP-15)) from the same breast cancer patients. The results presented here indicate that the oncoprotein SET is a useful biomarker for detecting CTCs, irrespective of EMT status, which may be beneficial for breast cancer treatment.

## Results

### Overexpression of SET in invasive breast carcinoma tissues

**Figure 1:**
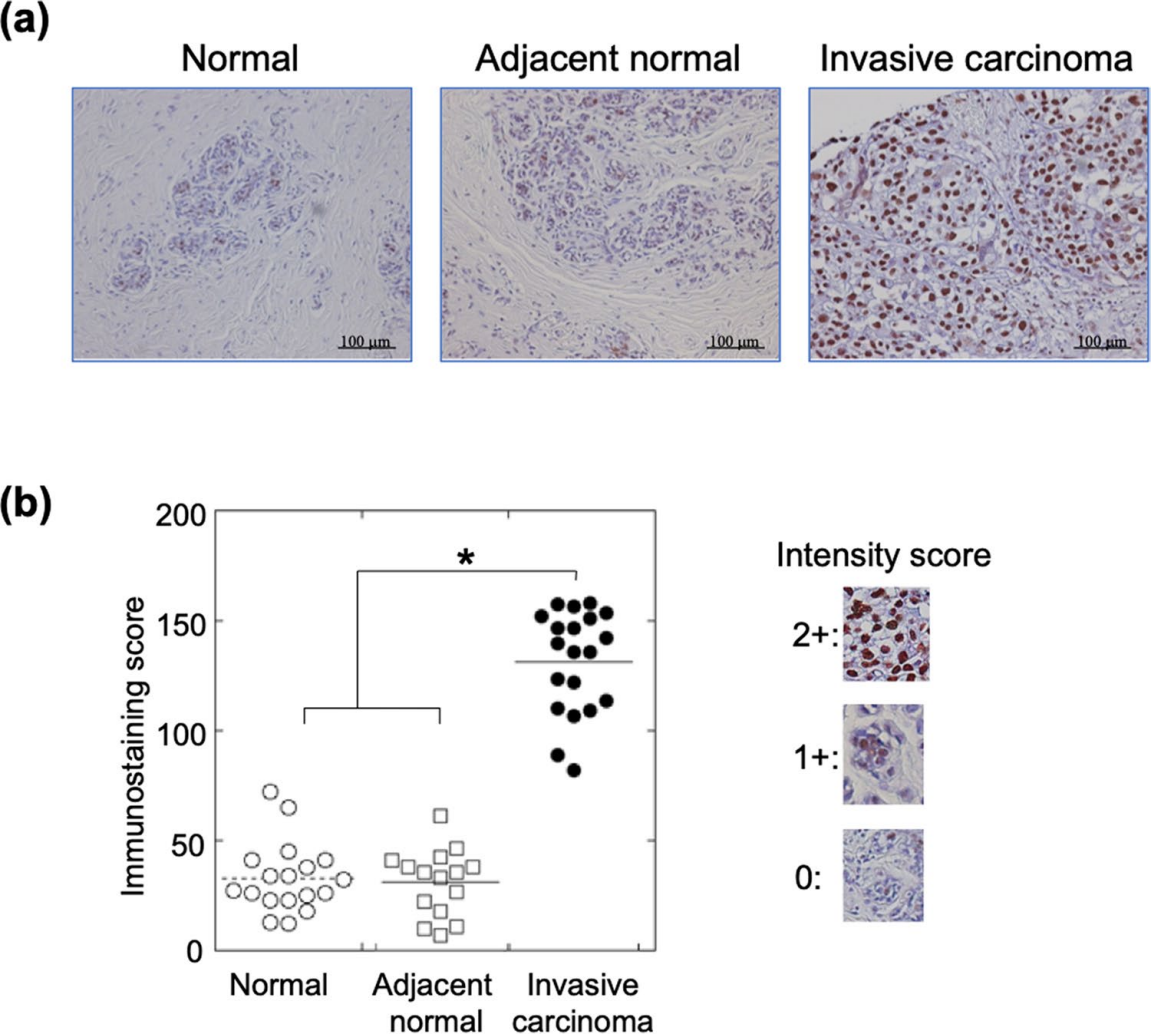
Immunohistochemical analysis of breast cancer tissue array. (**a**) Strong expression of SET was detected in nuclei of invasive carcinoma, but not in those of normal and adjacent normal tissues. (**b**) Immunostaining scores were determined as described in “Methods” section. Invasive carcinoma tissues showed significantly higher score than those of normal and adjacent normal tissues. *P < 0.01.

SET protein is a nuclear protein, and hence strong expression of SET was detected in the nuclei of invasive breast carcinoma tissues (Fig. 1a). Immunohistochemical staining of breast tissue array containing 24 cases each of normal tissue, adjacent normal tissue, and invasive ductal carcinoma revealed that the SET protein was significantly overexpressed in invasive carcinoma tissues compared to normal and adjacent normal tissues. Immunostaining scores of SET in normal, adjacent normal, and invasive carcinomas were 32.9 ± 15.9, 32.8 ± 18.7, and 148.1 ± 26.2, respectively (Fig. 1b). Next, we analysed the relationship between SET overexpression and breast cancer subtypes with varying expression of HR and HER2, using another breast cancer tissue array including 100 invasive carcinoma tissues from stage II–III breast cancers. As all invasive ductal carcinoma tissues were significantly stained with the anti-SET antibody, we classified them into three levels: level 1 (weak), level 2 (moderate), and level 3 (strong), corresponding to SET scores < 100, 100–130, and ≥ 130. More than 60% of all subtypes, including HR-positive/HER2-negative, HR/HER2-positive, HR-negative/HER2-positive, and triple-negative, showed strong level 3 SET expression (Table 1). In addition, we observed that higher SET expression was associated with higher Ki67 levels, indicating a correlation with high proliferation activity. Thus, the SET protein was overexpressed in invasive carcinoma irrespective of hormone receptor expression status.

**Table 1:** Overexpression of SET protein in invasive carcinomas with four clinical subtypes using breast cancer tissue microarray.

Characteristics	No. of samples	% of samples with SET levels			Average SET level	*P*
		1	2	3		
HR-positive/HER2-negative	46	13.0	21.8	65.2	2.5 ± 0.7	
HR/HER2-positive	19	5.3	10.5	84.2	2.8 ± 0.5	
HR-negative/HER2-positive	18	5.6	27.8	66.7	2.6 ± 0.6	
Triple negative	15	0.0	40.0	60.0	2.6 ± 0.5	
**Ki67**						
< 15%	73	9.6	28.8	61.6	2.5 ± 0.7	0.011
≥ 15%	25	4.0	8.0	88.0	2.8 ± 0.5	

### Contribution of SET to cell motility and stemness

**Figure 2:**
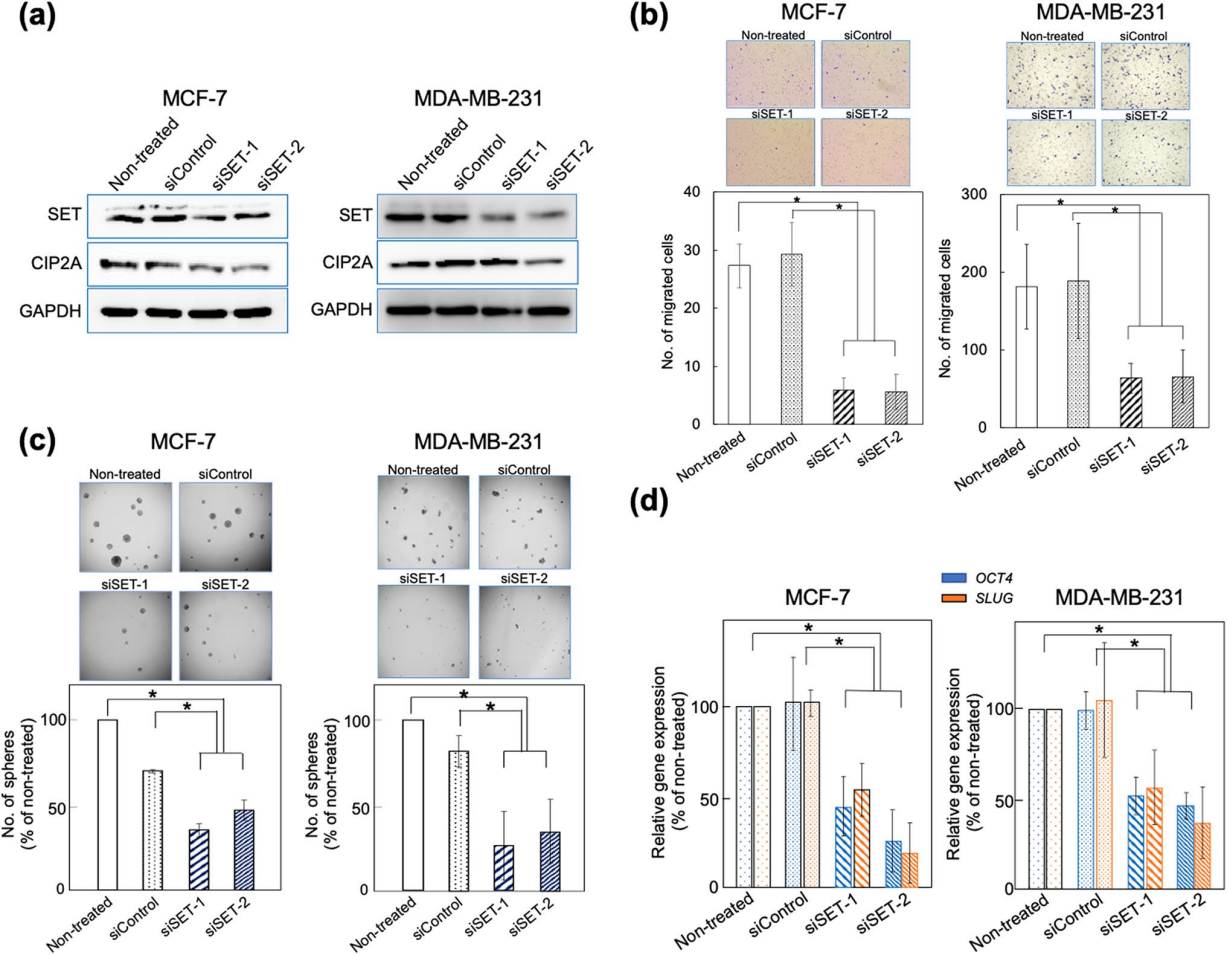
Knocking down of SET with siSETs reduced motility and mammosphere formation in MCF-7 and MDA-MD-231 cells. (**a**) Treatment with siSET-1 and siSET-2 reduced SET protein level, which was associated with reduction of CIP2A. Original full images in the figure were shown in Supplementary information, Figure S1. Due to cut the membrane prior hybridization or due to clear backgrounds, some of blot images have absent membrane edges, but specificities of bands were confirmed by knocking down using siRNAs. (**b**) Motility of MCF-7 and MDA-MD-231 cells was reduced upon SET knockdown. (**c**) SET knockdown inhibited mammosphere formation in MCF-7 and MDA-MB-231 cells. (**d**) *OCT4* and *SLUG* expression in mammospheres was suppressed by SET knockdown in MCF-7 and MDA-MB-231 cells. *P < 0.01.

SET protein is frequently overexpressed in various breast cancer cell lines including MCF-7 and MDA-MB-231^15^. Using MCF-7 cells as the HR-positive and epithelial type cell line, and MDA-MB-231 cells as the triple-negative and EMT-induced cell line, we analysed the effects of SET knockdown with SET-targeted siRNAs (siSET-1 and siSET-2) in transwell and mammosphere formation assays. Two SET protein bands were detected in MCF-7 and MDA-MB-231 cell lysates. The less abundant 41 kDa protein was confirmed as SET-α. Both cell lines expressed large amounts of SET-β and small amounts of SET-α. Treatment with siSET-1 and siSET-2 reduced both SET isomers protein levels by > 50% in both MCF-7 and MDA-MB-231 cells, but not after treatment with siControl (Fig. 2a). Similar to the results observed with SET knockdown, the level of CIP2A was also reduced by > 50% in both cell lines, suggesting that SET inhibition restored PP2A activity in combination with CIP2A downregulation. This is in agreement with the results of a previous report showing that targeting SET downregulates the *CIP2A* mRNA and increases PP2A activity in triple-negative breast cancer cells^26^. We observed that SET knockdown reduced the motility of cells using a transwell assay. Treatment with siSET-1 and siSET-2 reduced the number of migrated cells from 27.3 ± 3.8 to 6.0 ± 2.0 and 5.7 ± 3.1 (79.1% inhibition) in MCF-7 cells, and from 182 ± 54.5 to 64.0 ± 19.2 and 66.3 ± 34.0 (63.5% inhibition) in MDA-MB-231 cells (Fig. 2b). SET knockdown suppressed motility by > 60%, suggesting a significant role of SET overexpression in metastasis in breast cancer.

Furthermore, SET knockdown significantly suppressed mammosphere formation in MCF-7 and MDA-MB-231 cells. MCF-7 cells produced approximately 300 spheres with an average major axis of 150 μm for 2 weeks. Upon treatment with siSET-1 and siSET-2, the number of mammospheres reduced to 35.3% and 47.3% (64.7% and 52.7% inhibition), respectively; in contrast, treatment with siControl showed a weak reduction (Fig. 2c). Similarly, MDA-MB-231 cells formed about 250 spheres with an average major axis of 220 μm for 3 weeks. Treatment with siSET-1 and siSET-2 reduced mammosphere formation to 25.9% and 34.1% (74.1% and 65.9% inhibition), respectively. Furthermore, treatment with siSET-1 and siSET-2 significantly reduced the expression of *OCT4*, a stemness-related gene*,* and *SLUG*, an EMT-related gene, in mammospheres in both MCF-7 and MDA-MB-231 cells (Fig. 2d); however, *CD133*, *NANOG*, or *SOX2* expression was not altered (Supplementary Table S1). These results suggested that SET overexpression contributed to the maintenance of stemness and probably metastasis in both HR-positive and triple-negative breast cancers.

### Inhibition of tumour formation by MCF-7 cells following SET knockdown

**Figure 3:**
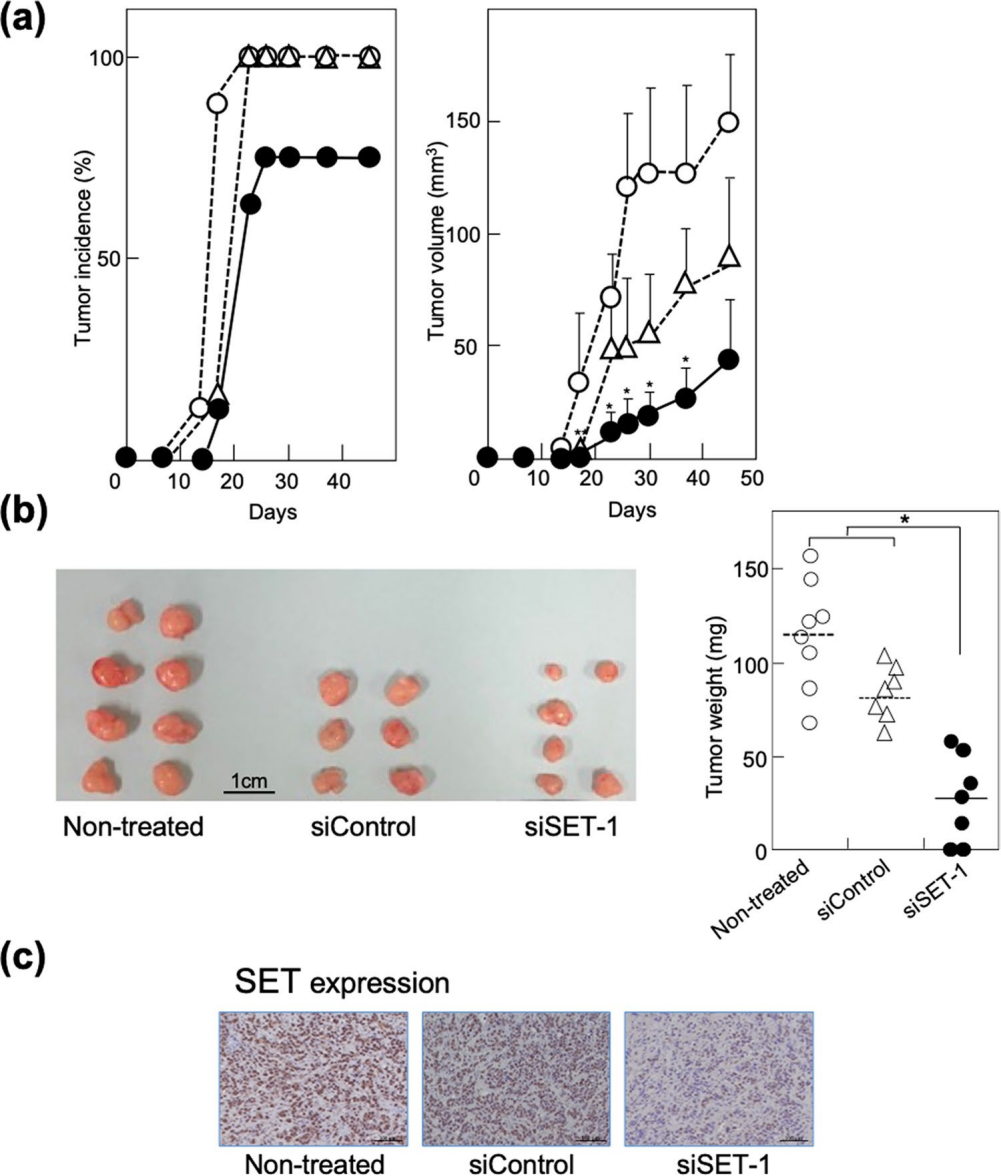
Knocking down of SET inhibited tumour formation of MCF-7 cells in SCID/Beige mice. (**a**) siSET-treated MCF-7 cells (●) showed lower incidence (right) and smaller tumours (left) than non-treated (○) and siControl-treated MCF-7 (△) cells. (**b**) The images show all tumours formed in non-treated (8 sites), siControl-treated (6 sites), and siSET-1-treated (8 sites) mice at the end of the experiment; the graph indicates the weight of each tumour in the three groups. (**c**) Immunohistochemical analysis showed that SET protein level was significantly lower in tumours developed in siSET-treated group than in those in the non-treated and siControl-treated groups. *P < 0.05.

Next, we examined the suppressive effect of siSET on tumour formation in vivo using a xenograft model of SCID/Beige mice. Non-treated, siControl-treated, and siSET-1-treated MCF-7 cells (5 × 10^6^ cells/site) were subcutaneously implanted at two sites in the flanks. At the end of the experiment on day 45, small tumours had developed at 75% (6/8) of the injected sites in the siSET-treated group, although tumours were found at 100% of the sites in the non-treated (8/8) and siControl-treated (6/6) groups (Fig. 3a,b). Tumour volume in the siSET-treated group (43.8 ± 11.9 mm^3^) was significantly smaller than that in the non-treated (149.9 ± 33.0 mm^3^) and siControl-treated (89.2 ± 37.9 mm^3^) groups. Tumour weight also decreased significantly from 115.1 mg ± 28.9 mg to 26.9 ± 23.5 mg (Fig. 3b). Immunohistochemistry confirmed the reduction in SET protein levels in tumours developed in the siSET-treated group: H-scores of tumours were 187.5 ± 52.0 for non-treated group, 198.3 ± 45.8 for siControl, and 66.7 ± 39.8 for siSET group, *P* < 0.05 (Fig. 3c). These results also indicated the contribution of SET to the progression of breast cancer.

### Significance of SET-expressing CTCs in patients with breast cancer

To examine whether SET can identify both epithelial and non-epithelial CTCs, we used a size-base microfluidic device, ClearCell FX, which successfully enriched heterogenous CTCs from blood samples of breast cancer patients^27^. Before assessing the presence of SET-expressing CTCs (SET-CTCs) in blood samples of patients with breast cancer, we confirmed that the size-based microfluidic device was more efficient in CTC enumeration than the CELLSEARCH system, which has been cleared by the Food and Drug Administration, USA^4^. The size-based device detected 3–12 epithelial-CTCs in 3.0 mL of blood in all five examined patients with advanced disease, whereas the CELLSEARCH system did not detect any CTCs in 7.5 mL of blood from the same patients. Furthermore, CTCs without epithelial features were also detected in two patients (Supplementary Table S2).

**Figure 4:**
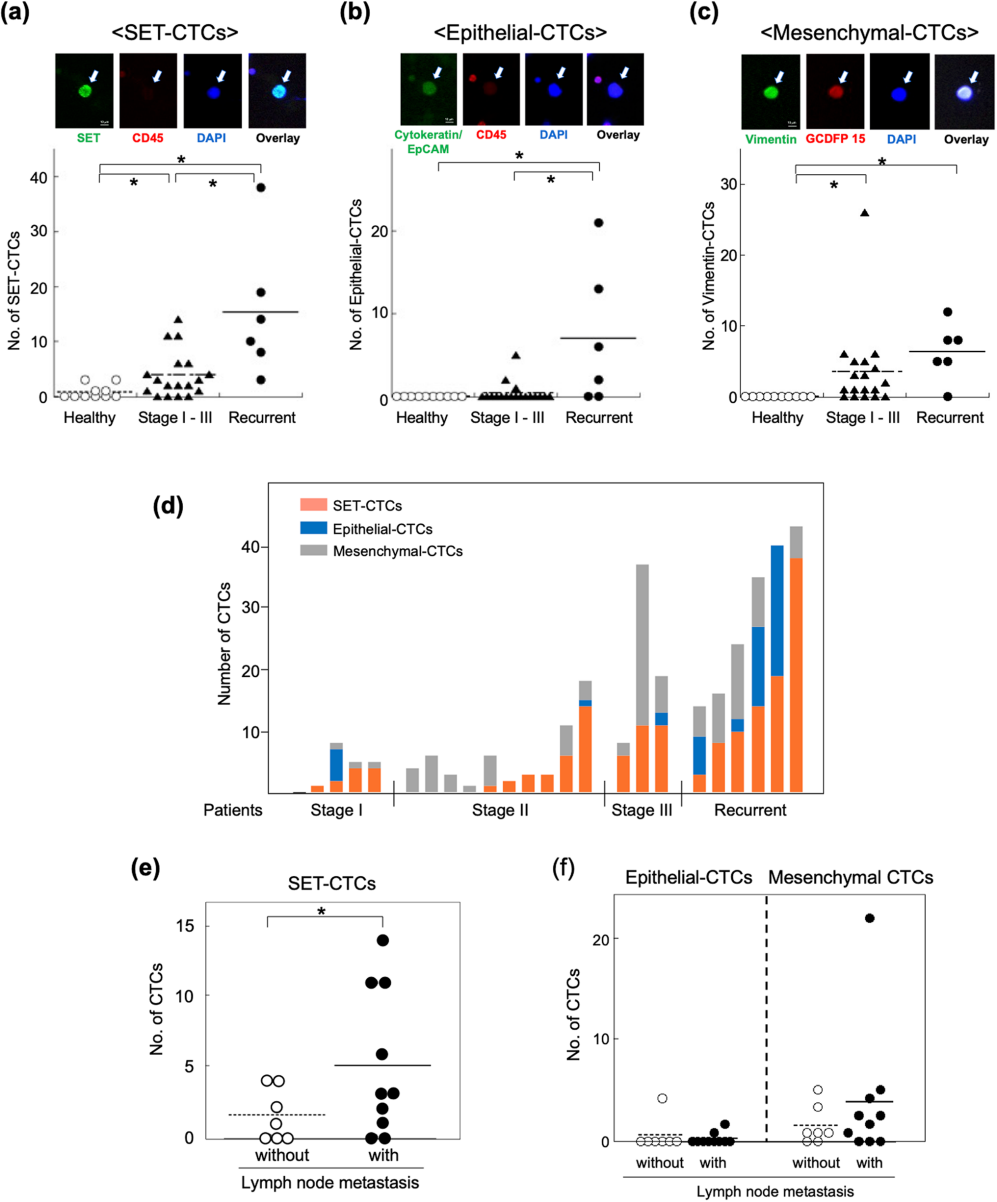
Immunocytochemical analysis of SET-CTCs, epithelial-CTCs and mesenchymal-CTCs. (**a**) Staining of the nucleus with anti-SET antibody (green), along with DAPI staining, but not with anti-CD45 antibody (absence of red). The number of SET-CTCs differed significantly between healthy control and patients with stage I–III disease, healthy control versus patients with recurrent disease, and patients with stage I–III disease versus patients with recurrent disease. (**b**) Representative staining of epithelial-CTCs with anti-pan-cytokeratin and anti-EpCAM antibodies (green), along with DAPI staining (blue), but not anti-CD45 antibody (absence of red). The number of epithelial-CTCs differed significantly between healthy control and patients with recurrent disease, and patients with stage I–III disease versus patients with recurrent disease. (**c**) Representative staining of a mesenchymal-CTC with anti-vimentin antibody (green) and anti-GCDFP15 antibody (red), along with DAPI staining (blue). The number of mesenchymal-CTCs differed significantly between healthy control and patients with stage I–III disease, and healthy control versus patients with recurrent disease. (**d**) SET-CTCs were detected not only in patients with epithelial-CTCs but also in those with mesenchymal-CTCs. Collared bars show the number of SET-CTCs (orange), epithelial-CTCs (blue), and mesenchymal-CTCs (gray) in each patient. (**e**) Patients with lymph node metastasis showed higher numbers of SET-CTCs than those with stage I–III disease. (**f**) No significant difference was observed in the number of epithelial- or mesenchymal-CTCs between patients with or without lymph node metastasis. *P < 0.05.

SET-CTCs were immunocytochemically analysed as nucleated cells (DAPI-positive) > 10 μm in diameter that stained with anti-SET (green) antibody, but not with anti-CD45 antibody (red) in patients with breast cancer (Fig. 4a). SET-CTCs were present in 13/18 (72.2%) stage I–III patients with a median value of 2.5 (2.5 cells/3 mL: range, 0–14), and 6/6 (100%) patients with recurrent disease with a median value of 12 (range, 3–38). In contrast, SET-CTCs were detected in 4/10 healthy controls with a median value of 0 (range, 0–3), indicating that the baseline of SET-CTCs may be 3 cells. When the cut-off value set to 4, the positivity of SET-CTCs was 0% for healthy controls, 44.4% for patients in stage I–III, and 83.3% for patients with recurrent disease (Table 2). The number of SET-CTCs and positivity of patients in stage I–III disease and recurrent disease were significantly higher than those in the healthy controls (*P* < 0.05) (Table 2, Fig. 4a). Furthermore, the number of CTCs in patients with recurrent disease was significantly higher than that in patients with stage I–III disease. To understand the relationship between SET-CTCs and other CTC subpopulations, including CTCs with epithelial and mesenchymal features, we examined the numbers of epithelial-CTCs, which are pan-cytokeratin-and EpCAM-positive and CD45-negative, and mesenchymal-CTCs, which are vimentin-positive and GCDFP-15-positive in the same patients, as described in “Methods” section. Epithelial-CTCs were detected in 3/18 patients with stage I–III disease (16.7%) with a median value of 0 (range, 0–5), and in 4/6 (66.7%) patients with recurrent breast cancer with a median value of 4 (range, 0–21) (Table 2, Fig. 4b). In contrast, mesenchymal-CTCs were detected in 13/18 (72.2%) patients with stage I–III disease with a median value of 1.5 (range, 0–26), and in 5/6 (83.3%) patients with recurrent disease with a median value of 6.5 (range, 0–12) (Table 2, Fig. 4c). As shown in Fig. 4d, all 7 patients with epithelial-CTCs also harboured SET-CTCs, and 6 patients harboured mesenchymal-CTCs. Among 18 patients with mesenchymal-CTCs, 14 were positive for SET-CTCs. These results suggested that SET is widely expressed in both epithelial- and mesenchymal-CTCs. In this study, most patients were HR-positive/HER2 negative. Three patients with recurrent disease and one stage I-III patients were triple-negative. SET-CTCs were found in all four triple-negative patients. However, epithelial-CTCs were not found in the triple-negative stage I-III patient.

**Table 2:** Numbers of SET-CTCs, epithelial-CTCs, and mesenchymal-CTCs in breast cancer patients and healthy controls along with their characteristics. *P<0.05, **P<0.01.

	Breast cancer patients		Healthy controls
	Stage I–III	Recurrent	
Number of samples	18	6	10
Age	52.5 ± 13.7 (37–87)	60.0 ± 10.0 (43–69)	36.2 ± 14.1 (21–64)
**Lymph node metastasis**			
Yes	10	–	–
No	7		
**SET-CTCs**			
Samples with ≥ 1	13 (72.2%)	6 (100%)**	4 (40.0%)
Samples with ≥ 4	8 (44.4%)*	5 (83.3%)**	0
Median (range)	2.5* (0–14)	12** (3–38)	0 (0–3)
**Epithelial-CTCs**			
Samples with ≥ 1	3 (16.7%)*	4 (66.7%)**	0
Median (range)	0* (0–5)	4** (0–21)	0
**Mesenchymal-CTCs**			
Samples with ≥ 1	13 (72.2%)*	5 (83.3%)**	0
Median (range)	1.5* (0–26)	6.5* (0–12)	0

Next, we analysed the relationship between the number of SET-CTCs and lymph node metastasis among patients with stage I–III disease, and observed that patients with lymph node metastasis showed a significantly higher number of SET-CTCs than patients without lymph node metastasis; the median numbers were 3 and 1, respectively (Fig. 4e). Unlike in SET-CTCs, no correlation was observed between lymph node metastasis and numbers of epithelial-CTCs or mesenchymal-CTCs (Fig. 4f). Thus, although the sample number size was small, our results clearly indicate that SET is a promising biomarker for detecting CTCs in patients with various subtypes of breast cancer, which can be used for early monitoring of metastasis.

## Discussion

Here, we report for the first time that SET is a possible biomarker for CTCs, and it can be used for the detection of metastasis in patients with breast cancer irrespective of the clinical subtype. Immunohistochemical analysis of tissue microarrays, as well as in vitro and in vivo studies using siSETs indicated that overexpression of SET plays a significant role in progression, especially motility and stemness in breast cancer. Our results are consistent with previous reports showing that knockdown of SET with siRNA significantly reduces tumour growth of MDA-MB-231, MDA-MB-436, and MDA-MB-468 in xenograft experiments in non-obese diabetic/SCID/γ-chain null mice^15^. Furthermore, SET overexpression is associated with worse recurrence-free survival and it has been shown to reduce tamoxifen-induced anti-tumour effect in patients with primary breast cancer^29^. High SET protein levels are also associated with poor prognosis of metastatic colon cancer, non-small-cell lung cancer, pancreatic cancer, gastric cancer, liver cancer, CML, AML, and lymphoma^16–22^. SET enhances cell migration, colony-forming activity, and EMT transition in colorectal cancer^30^. High expression of SET is shown to contribute to the progression of various types of cancer, suggesting the usefulness of SET as a biomarker of CTCs in other cancers in addition to breast cancer. Therefore, we suggest that SET is a promising non-specific diagnostic marker for monitoring disease progression and treatment response for various cancers, rather than a specific biomarker for breast cancer.

In this study, we used the size-based microfluidic device as a label-free method, which is based on differences in the mechanical features, such as the size of tumour cells and white blood cells (tumour cells are larger than white blood cells)^27^. Various methods have been developed for isolation of CTCs based on their physical properties, biological characteristics, and tumour-specific surface proteins^31^. Among all of them, the method used in this study is one that can enumerate heterogenous CTCs^28^. In the analysis of epithelial-CTCs, large cells were found to be negative for epithelial marker and CD45. We think that some of the large cells were SET-CTCs and mesenchymal-CTCs. In addition, some SET-CTCs were positive for epithelial or mesenchymal markers. Therefore, we believe that CTCs can be detected in patients with undetectable epithelial-CTCs and mesenchymal-CTCs using SET as a biomarker. The number of SET-CTCs in patients with recurrent disease was significantly higher than that in patients with stage I–III disease, and a high number of SET-CTCs were associated with the presence of lymph node metastasis in patients with stage I–III breast cancer. One limitation of the study is that the number of examined patients was small. And, few SET-positive large cells were detected in the healthy controls. As SET is a ubiquitously expressed protein, we need to improve the specificity of the method used for SET-CTC analysis. We think that further follow-up of patients with early-stage breast cancer is required to determine a cut-off value for detection of early metastasis. In our study, we observed 14 SET-CTCs/3 mL of the peripheral blood of a 37-year-old patient with stage II breast cancer, who had axillary lymph node metastases and multiple tumours in the ipsilateral breast. This patient showed the highest number of SET-CTCs among stage I-III patients and the number higher than even the median value of patients with recurrent disease. We believe that combination analysis with SET, epithelial and mesenchymal markers will improve the clinical significance of CTC analysis by increasing sensitivity and specificity. Ito et al. reported that determining the numbers of both mesenchymal- and epithelial-CTCs might predict survival for breast cancer patients receiving eribulin^32^. Furthermore, analysis of SET-CTCs will enable real-time monitoring of treatment response to therapeutic drugs. We performed a preliminary CTC analysis to compare CTCs numbers before and after treatment in three patients with breast cancer. Two patients treated with eribulin showed a clear reduction in the number of SET-CTCs (from 8 to 0 and from 10 to 1); while the patient treated with conventional chemotherapy did not (from 14 to 22). Although their clinical response has to be confirmed, this pilot study shows the possibility of monitoring therapy response using SET-CTCs.

There are two major isoforms of SET, SET-α and SET-β, and SET-β is reported to be more widely expressed compared with SET-α. In MCF-7 and MDA-MB-231 cells, we detected a major band with 39 kDa and a minor band with 41 kDa using a pan-SET antibody. A monoclonal antibody for specific SET-a reacted with the protein corresponding to the minor band. Therefore, we suggest that low amounts of SET-α and high amounts of SET-β are expressed in both cell lines. Recently, distinctions in transcriptional regulation and function between two isoforms have been reported. Since we used a pan-SET antibody in this CTC study, further investigations using specific antibodies for SET-α and SET-β will provide more insights into the significance of SET isomers in cancer progression.

SET directly binds to the catalytic subunit of PP2A and inhibits its phosphatase activity^10^. Therefore, SET is a potent inhibitor of protein dephosphorylation among PP2A inhibitors and inhibition of SET with siRNA strongly impacts targeted peptides^25^. As PP2A is a critical negative regulator of various pathways related to MYC, Wnt, extracellular signal-regulated kinase (ERK), and Akt, SET overexpression induces stabilisation and activation of c-Myc transcriptional activity and related signalling pathways^15,33^. Furthermore, SET maintains cancer cell stemness by stabilizing phosphorylated E2F1 via inhibition of PP2A in gastric cancer^34^, and stable SET knockdown inhibits migration and invasion of breast cancer cells via reduction of matrix metalloproteinase 9 (MMP-9)^35^. Although we did not investigate whether c-Myc, E2F1, or MMP-9 were inhibited by SET knockdown in breast cancer cells in this study, we observed that treatment with siSET-1 and siSET-2 significantly reduced phosphorylation of Akt in MCF-7 cells (data not shown), suggesting the involvement of the Akt signalling pathway. Therefore, SET plays a key role in malignant progression in breast cancer of all subtypes. These results suggest that SET is a proper target to inhibit multiple pathways involving proliferation, motility, invasion, and stemness, which result in tumour promotion, progression, and metastasis.

Recently, reactivation of PP2A activity using small molecules that either antagonise its inhibitors (SET and CIP2A), or directly activate PP2A, has attracted attention as a new strategy for cancer therapy^14,33^. These are named PP2A activating drugs. Several SET-targeting agents have been developed. For example, COG112, anapolipoprotein E-mimetic peptide interacting with SET, inhibited the motility of various cancer cells^33^; FTY720, a synthetic sphingosine analogue, binds to SET, leading to PP2A activation and anti-tumour activity in lung cancer and colorectal cancer^24,36^; and OP449 (COG449), a synthetic peptide, directly binds to SET and/or interferes with its PP2A-inhibition function^37^. OP449 showed anti-leukemic activity toward tyrosine kinase inhibitor (TKI)-resistant CML and AML in primary patient samples. Notably, combination of TKI and OP449 synergistically reduced the viability and clonogenic potential of leukemic cell lines and primary CD34^+^ CML progenitors^38^. The combined effects of an anticancer agent, such as trastuzumab, and SET-targeting drugs on breast cancer patients should be investigated in future.

Based on our results, we concluded that analysis of SET-CTCs will provide new insights regarding the mechanism of metastasis, and useful information for treatment design using SET-targeting drugs in near future.

## Methods

### Cell culture and reagents

Human breast cancer cell lines, MCF-7 and MDA-MB-231, were purchased from the American Type Culture Collection (Manassas, VA, USA). The cells were grown in Roswell Park Memorial Institute (RPMI) 1640 medium containing 10% foetal bovine serum (FBS; Sigma-Aldrich, MO, USA) at 37 °C in the presence of 5% CO_2_. The cells, which were confirmed to be mycoplasma-free, were used at less than 20 passages and were authenticated using short-tandem repeat profiling by ATCC. Anti-SET, anti-SET-α (ab1183), and anti-gross cystic disease fluid protein-15 (GCDFP-15) antibodies were purchased from Abcam Plc, (Cambridge, UK), and anti-CIP2A and anti-vimentin antibodies were from Santa Cruz Biotechnology, Inc. (Danvers, MA, USA). Anti-YAP, anti-pan-keratin (CK4, 5, 6, 8, 10, 13, 18), anti-EpCAM, and anti-CD45 antibodies were obtained from Cell Signaling Technology (MA, USA). AF647-anti-cytokeratin (pan-reactive, BioLegend, CA, USA), PE-anti-CD45 (Abnova Co., Taipei, Taiwan), anti-cytokeratin C-36H5 (CK 7, 8, 18, 19, MiltenyiBiotec GmbH, Gladbach, Germany), anti-glyceraldehyde 3-phosphate dehydrogenase (GAPDH) (Trevigen, MD, USA), AF594 anti-rabbit IgG, and AF488 anti-mouse IgG (Life Technologies, CA, USA) were used for the experiments.

### Immunohistochemistry

Human paraffin embedded tissue arrays were purchased from US Biomax, Inc. (Rockville, MD, USA). Breast cancer and adjacent normal tissue array (BR724), and breast carcinoma with breast tissue microarray (BC08116d) were used. The tissue arrays were incubated with anti-SET antibody (1:800) for 40 min at 37 °C after blocking with Protein Block (DAKO, Tokyo, Japan), and the secondary antibody (*N*-Histofine simple stain MAX-PO (Multi), Nichirei Bioscience Inc. Tokyo, Japan) was applied as described previously^39^. The stained tissues were observed and photographed at equivalent to 40 × using NanoZoomer-XR and NDP.view2 (Hamamatsu Photonics K.K., Shizuoka, Japan). The relative staining intensity of SET in breast tissues was reviewed by two independent individuals. The score was calculated based on the staining intensity as percentage of stained cells × intensity score.

### Knockdown of SET with siRNA

SET-targeted siRNAs (siSET-1 and siSET-2) and a control siRNA were purchased from OriGene Technologies, Inc. (Rockville, MD, USA). The sequences of siSET-1 and siSET-2 were 5′-GGTCTCTGTTTAGTAAATACATCAC-3′ and 5′-GCTATGTGGATTGAGTAATGGGATT-3′, respectively. siRNA (10 nM) was transfected into MCF-7 cells using Lipofectamine 3000 (Invitrogen, CA, USA), and in MDA-MB-231 cells using Lipofectamine RNAiMAX (Invitrogen)^40^. Western blot analysis, mammosphere formation, and transwell assay were conducted after 2 days.

### Western blotting

Whole cell lysates were obtained using a lysis buffer, as described previously^41^. The cell lysates (10 μg) were subjected to sodium dodecyl sulphate–polyacrylamide gel electrophoresis (SDS–PAGE) and transferred onto a nitrocellulose membrane. The membranes were blocked with 3% skim milk in 20 mM Tris-HCl (pH 7.6) containing 150 mM NaCl with 0.1% Tween 20, and then incubated with the primary antibody overnight at 8 °C. After incubation with horseradish peroxidase-conjugated secondary antibody against rabbit IgG (GE Healthcare, UK) or goat IgG (Santa Cruz, CA, USA), the bound antibody was detected using ImmunoStar LD (Wako Pure Chemical Industries, Ltd., Tokyo, Japan) and a C-DiGit Blot Scanner (LI-COR Biosciences Inc., NE, USA), as described previously^41^. At least three independent experiments were conducted.

### Transwell assay

Cell motility was determined using a Transwell cell culture chamber (Becton–Dickinson, NJ, USA). Cells (1 × 10^4^) in serum-free RPMI 1640 medium were added to the upper chamber and incubated for 6 h with 1% FBS in the lower chamber. The cells that migrated to the other side of the filter with pore size of 8 μm were stained with 0.4% trypan blue and counted, as described previously^40^. The data represent an average of three independent experiments that were conducted in duplicate.

### Mammosphere formation assay

MCF-7 (100 cells/well) or MDA-MB-231 cells (500 cells/well) were seeded into ultra-low-attachment 96-well plates (Corning Inc., Corning, NY, USA) and grown in Dulbecco’s modified Eagle’s medium/F12 medium (Gibco, Rockville, MD) containing 0.45% methylcellulose, 50 ng/mL epidermal growth factor, 50 ng/mL fibroblast growth factor (PeproTech, USA), and B27 supplement (Invitrogen) for 2 or 3 weeks. Each treatment was conducted in six wells. All wells were imaged using an All-in-One fluorescence microscope (BZ-X700, Keyence, Tokyo, Japan), and the number of spheres measuring > 100 µm in a minor axis were counted^41^. Three independent experiments were conducted.

### Quantitative reverse transcription RT-PCR (qRT-PCR)

The mammospheres were collected using a filter with pore size of 77 μm (Spheroid Catch, Watson Co. Ltd., Tokyo, Japan) and total RNA was isolated using Isogen (Nippon Gene Co. Ltd., Toyama, Japan). Total RNA was reverse transcribed into cDNA using oligo(dT)_16_ and MuLV reverse transcriptase (Thermo Fisher Scientific, Tokyo, Japan), and real-time PCR was conducted using SYBR Green I (LightCycler 480, Roche Life Science, Basel, Switzerland), as described previously^41^.

### Tumour xenograft experiments

All animal experimental protocols were approved by the Animal Care and Use Committee of Saitama University (H30-A-1-12). All methods were carried out in accordance with the guidelines for the Care and Use of Experimental Animals of Saitama University. This in vivo study was carried out in compliance with the ARRIVE guidelines. Five-week-old female SCID/Beige mice were obtained from Charles River Laboratories Japan, Inc., Kanagawa, Japan, and randomly divided into non-treated, siControl-treated, and siSET-treated groups (3 or 4 mice/group). After treatment with siSET-1 or siControl for 2 days, 5 × 10^6^ cells/100 μL saline of MCF-7 cells were injected at two sites of the subcutaneous flanks of mice. Body weight were monitored weekly, and tumour size was measured using a caliper gauge every 3–4 days for 46 days. Tumour volume was calculated using the formula width^2^ × length/2^40^. Tumour weight were determined after dissection of tumour tissues from euthanized mice with pentobarbital sodium solution (64.8 mg/mL, Kyoritsu Seiyaku Co., Tokyo) at the study end-point.

### Enumeration of CTCs from peripheral blood and immunocytochemistry

Blood samples were obtained from 29 patients with breast cancer and 10 healthy volunteers who were enrolled between December 2016 and March 2020. Informed signed consent was obtained from patients and healthy volunteers before blood collection, and the study was approved by the ethics committees of the Saitama Cancer Center (H26-423, H29-669) and Saitama University (H26-21, R1-E-2). All methods were performed in accordance with relevant guidelines and regulations of the Saitama University and the Saitama Cancer Center. Blood samples were collected in sterile EDTA-coated vacutainer tubes.

CTCs in blood were enumerated using ClearCell FX (Clearbridge BioMedics, Pte Ltd., Singapore) according to the manufacturer’s instruction^27^. After treatment with a red blood cell lysis buffer, the nucleated cell fraction from 7.5 mL blood was loaded on the CTChip. The enriched CTCs were collected via centrifugation at 500 × *g* for 15 min at room temperature. This enumeration was conducted twice per individual, and the total CTCs-enriched fraction was divided into four samples to analyse SET-, epithelial-, and mesenchymal-CTCs using immunocytochemical staining.

The CTCs-enriched fractions were fixed with 4% paraformaldehyde in phosphate buffered saline (PBS) containing 0.1% Triton X-100 for 10 min. Immunocytochemical staining was conducted after blocking with 5% FBS and 5% human FcR blocking reagent (MiltenyiBiotec GmbH) in PBS for 30 min. For SET-CTCs analysis, the CTCs were incubated with anti-SET and anti-CD45 antibodies for 30 min, and then stained with appropriate fluorescently labelled secondary antibodies, along with 4′6-diamidino-2-phenylindole (DAPI). SET-positive (green), CD45-negative, and DAPI-positive cells more than 10 μm in diameter were designated as SET-CTCs. For epithelial-CTCs, an anti-cytokeratin and EpCAM antibody cocktail including anti-pan cytokeratin, anti-cytokeratin (C-36H5), anti-EpCAM, and anti-CD45 antibodies were used. For mesenchymal-CTCs, anti-vimentin and anti-GCDFP 15 antibodies were used, and vimentin-positive (green) and GCDFP-15-positive (red) cells were designated as mesenchymal-CTCs. The size and staining intensity of CTCs were analysed using Hybrid Cell Count (BZ-H3A) under an All-in-One fluorescence microscope BZ-X700 (Keyence Co., Osaka, Japan).

### Statistical analysis

Statistical analyses for in vitro and in vivo studies were performed using one-way analysis of variance (ANOVA), followed by Dunnett’s test. Statistical tests in CTCs analysis were performed using SPSS ver. 26 (SPSS Inc. USA). Each experiment was conducted independently at least thrice, and values are expressed as mean ± standard deviation (SD). The association between CTC numbers and clinical parameters was assessed using Student’s *t*-test. *P* < 0.05 was considered statistically significant.

## Supplementary information

Supplementary Information.

## Data Availability

The datasets generated during and/or analysed during the current study are available from the corresponding author on reasonable request.
