# Supplementary material for: Deregulation of protein phosphatase 2A inhibitor SET is associated with malignant progression in breast cancer
Source: Sci Rep. 2021 Jul 9;11:14238. doi: 10.1038/s41598-021-93620-y (PMC8270961; doi:10.1038/s41598-021-93620-y)

Supplementary information

Deregulation of protein phosphatase 2A inhibitor SET is associated with malignant progression in breast cancer

Katsunori Tozuka[1†], Pattama Wongsirisin[2,3†], Shigenori E Nagai[4], Yasuhito Kobayashi[5], Miki Kanno[2,3], Kazuyuki Kubo[1], Ken Takai[4], Kenichi Inoue[4], Hiroshi Matsumoto[1], Yoshihito Shimizu[5] & Masami Suganuma[2,3]*

Table S1. Specific inhibition of *OCT4* and *SLUG* gene expression in mammospheres by treatment with siSETs in MCF7 and MDA-MB-231 cells

	Relative expression (fold of non-treated)**					
	MCF-7			MDA-MB-231		
	siControl	siSET-1	siSET-2	siControl	siSET-1	siSET-2
Stemness genes						
CD133	1.21 ± 0.34	1.31 ± 0.37	0.55 ± 0.10	ND	ND	ND
NANOG	1.06 ± 0.31	1.50 ± 0.44	0.94 ± 0.21	0.73 ± 0.25	0.66 ± 0.52	1.43 ± 0.25
SOX2	1.32 ± 0.14	1.01 ± 0.26	0.96 ± 0.25	1.01 ± 0.01	1.20 ± 0.71	1.04 ± 0.58
OCT4	0.99 ± 0.11	0.52 ± 0.10*	0.46 ± 0.07*	1.02 ± 0.26	0.57 ± 0.06*	0.26 ± 0.18*
ALDH1A1	ND	ND	ND	2.02 ± 1.38	3.52 ± 0.62	3.65 ± 1.79
EMT marker genes						
SLUG	1.04 ± 0.32	0.55 ± 0.21*	0.46 ± 0.07*	1.02 ± 0.07	0.54 ± 0.15*	0.20 ± 0.17*

*: P<0.05

**: Expression level of each gene normalized by α-tubulin in non-treated cells was expressed as 1.0.

ND means not detected

Table S2. Comparison of efficiency of CTCs enumeration between the size-based microfluidic device and CellSearch™ system

Patients	Size-based microfluidic device (No. of CTCs/3.0 mL)		CellSearch™ System (No. of CTCs/7.5 mL)
	Epithelial-CTCs*	Non-epithelial CTCs**	
P1	3	0	0
P2	8	0	0
P3	12	1	0
P4	7	0	0
P5	7	2	0

*Epithelial-CTCs were defined as CK/EpCAM-positive, CD45-negative and DAPI-positive.

**Non-epithelial-CTCs were defined as CK/EpCAM-negative, CD45-negative and DAPI-positive.

Supplemental Figure legends

Figure S1. Original full western blot images and gel bolts utilized in Figure 2a

Figure S2. Other replicates of western blot images (N-2 & N-3)

Figure S1- SET of MCF-7- Original

Figure 2 (a)

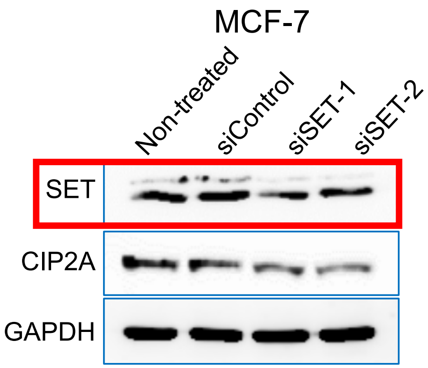

<Original image>

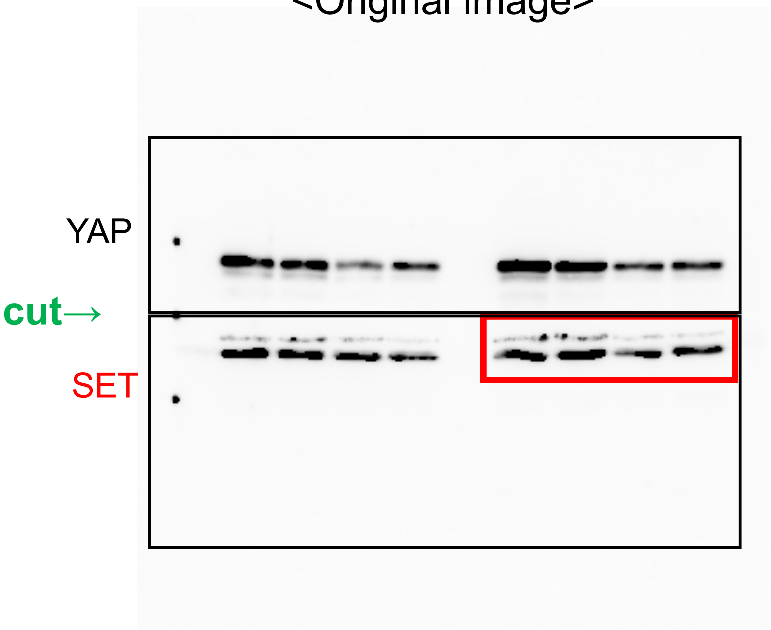

We cut the membrane prior to hybridization. All blots are indicated, but membrane edges are not clear. So, all other replicates are showed as follows. Dark image of N-2 shows membrane edges.

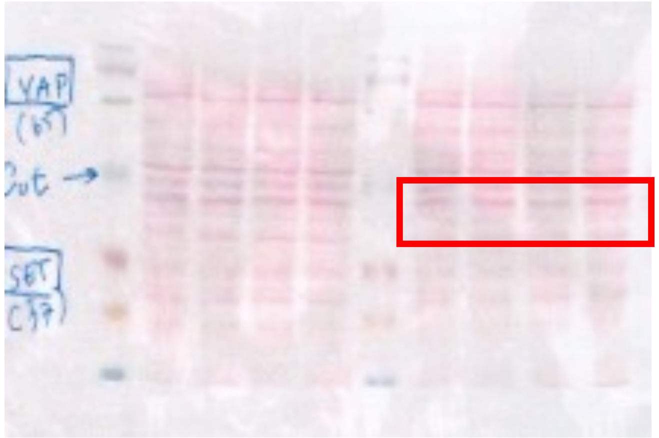

Figure S1- CIP2A of MCF-7 - Original

Figure 2 (a)

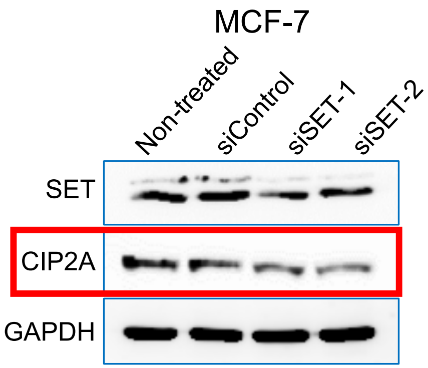

<Original image>

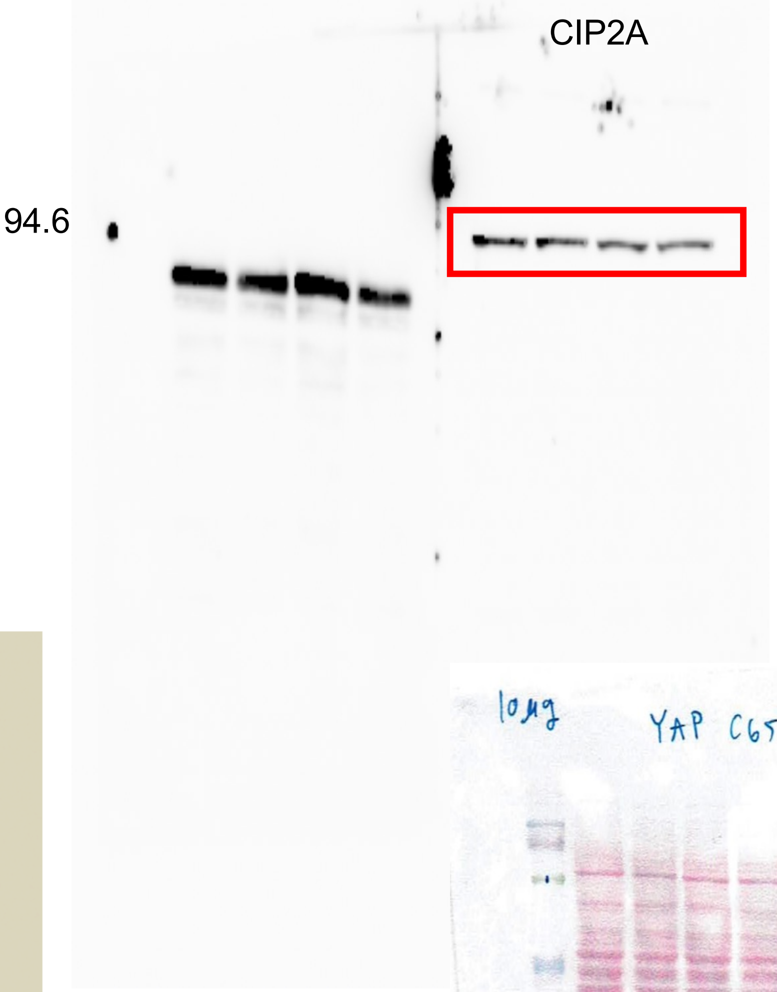

Dark image showing membrane edges.

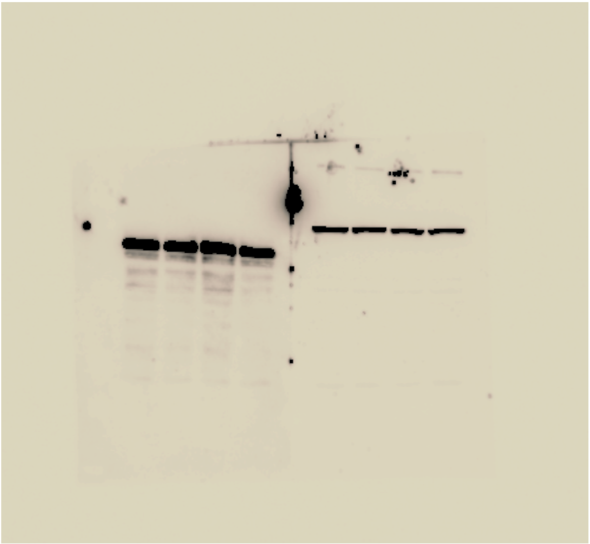

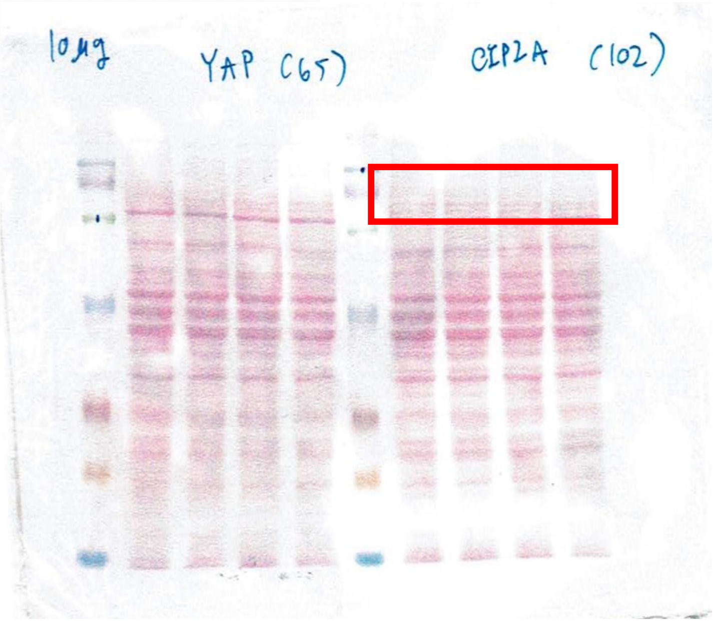

Figure S1- GAPDH of MCF-7 - Original

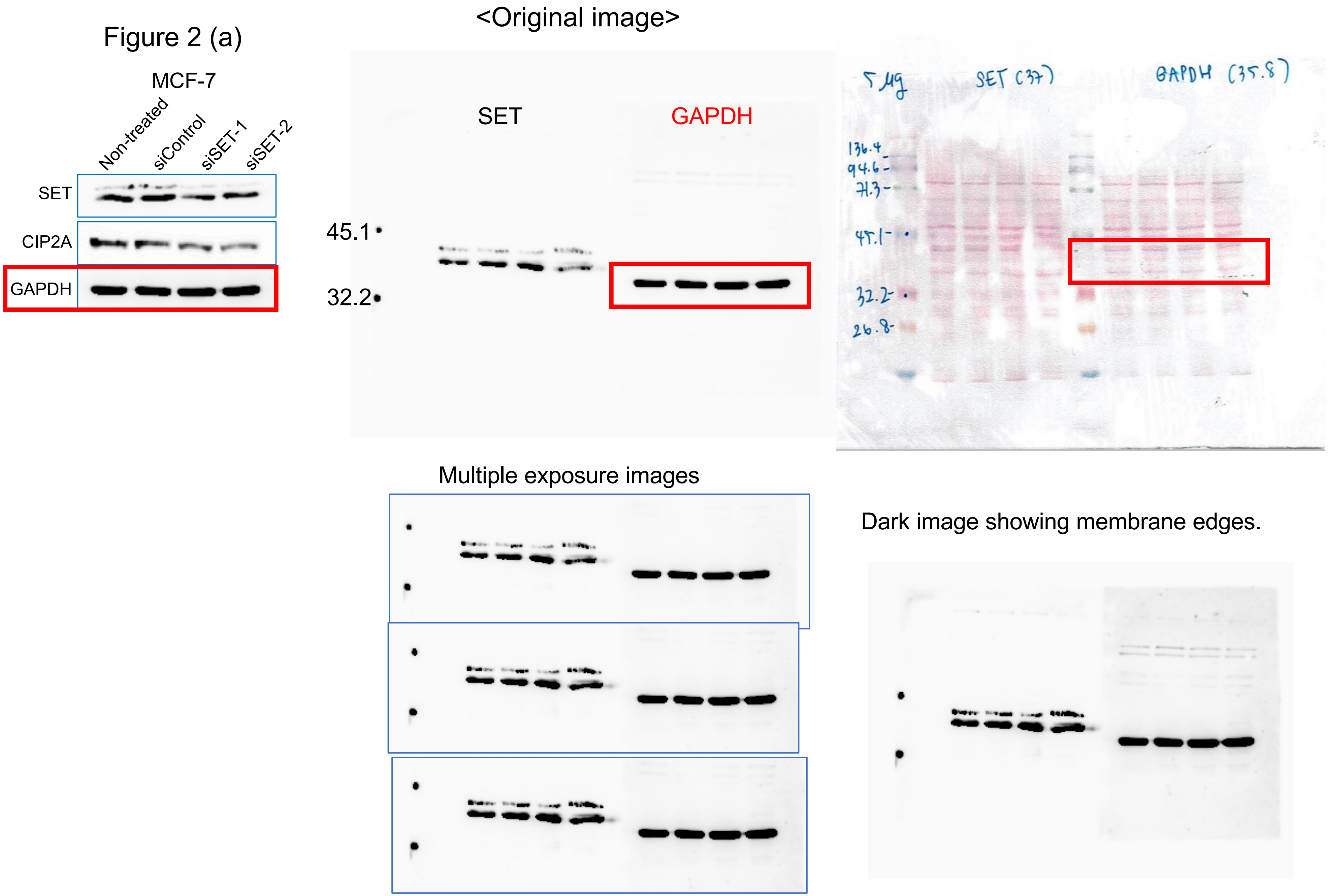

Figure S1- SET of MDA-MB-231 - Original

Figure 2 (a)

MDA-MB-231

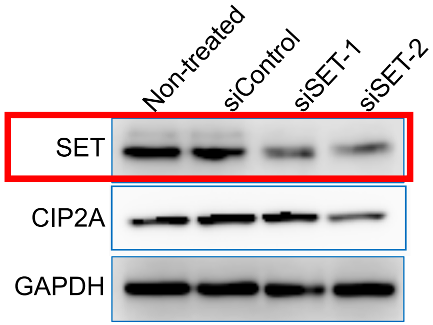

<Original image>

SET

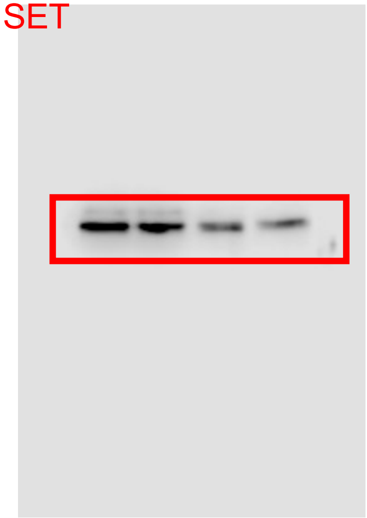

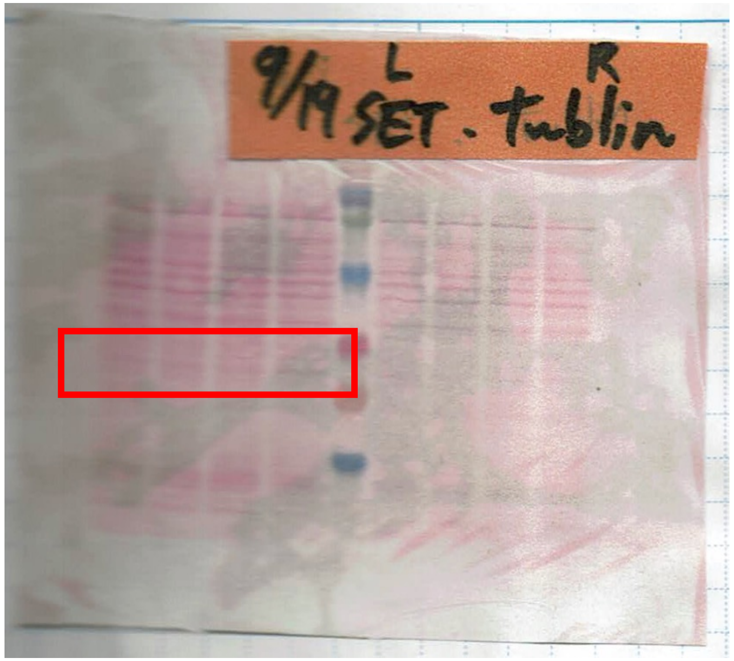

We did not cut the membrane prior to hybridization. But membrane edges are not clear, due to clear background.

Figure S1- CIP2A of MDA-MB-231 – Original

Figure 2 (a)

MDA-MB-231

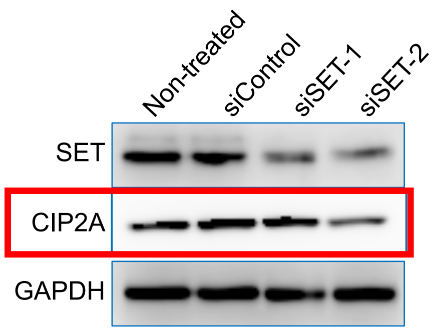

<Original image>

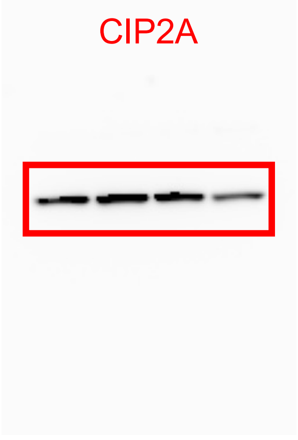

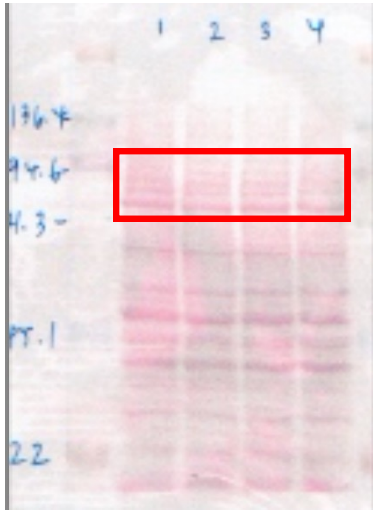

We did not cut the membrane prior to hybridization. But edges are not clear due to clear background.

Figure S1- GAPDH of MDA-MB-231 - Original

Figure 2 (a)

MDA-MB-231

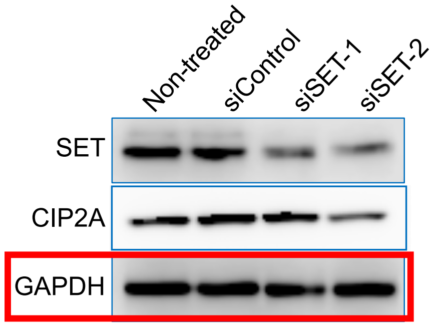

<Original image>

GAPDH

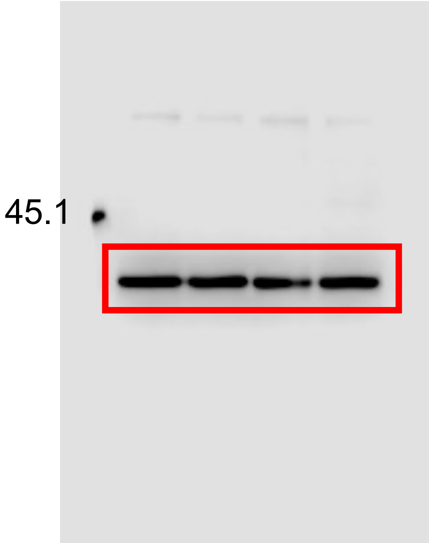

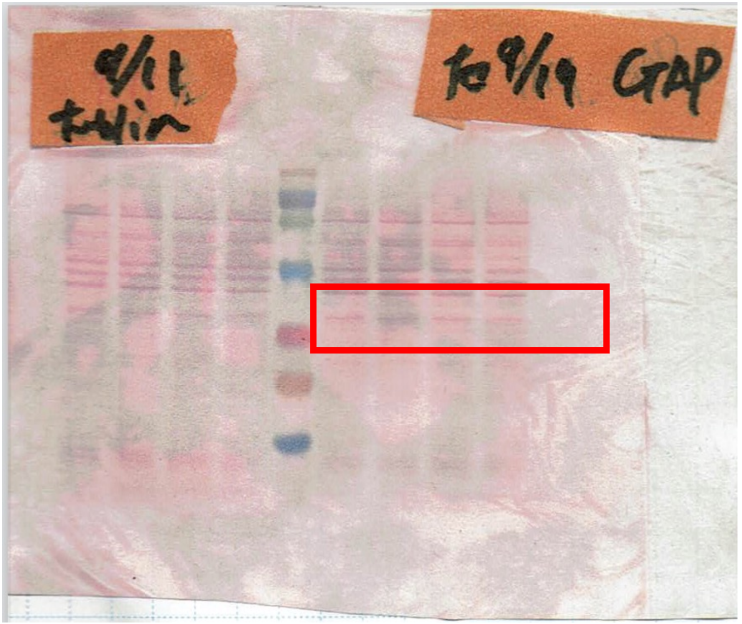

We did not cut the membrane prior to hybridization. But, membrane edges are not clear.

Figure S2- SET of MCF-7- other replicates (N-2 & N-3)

<N-2>

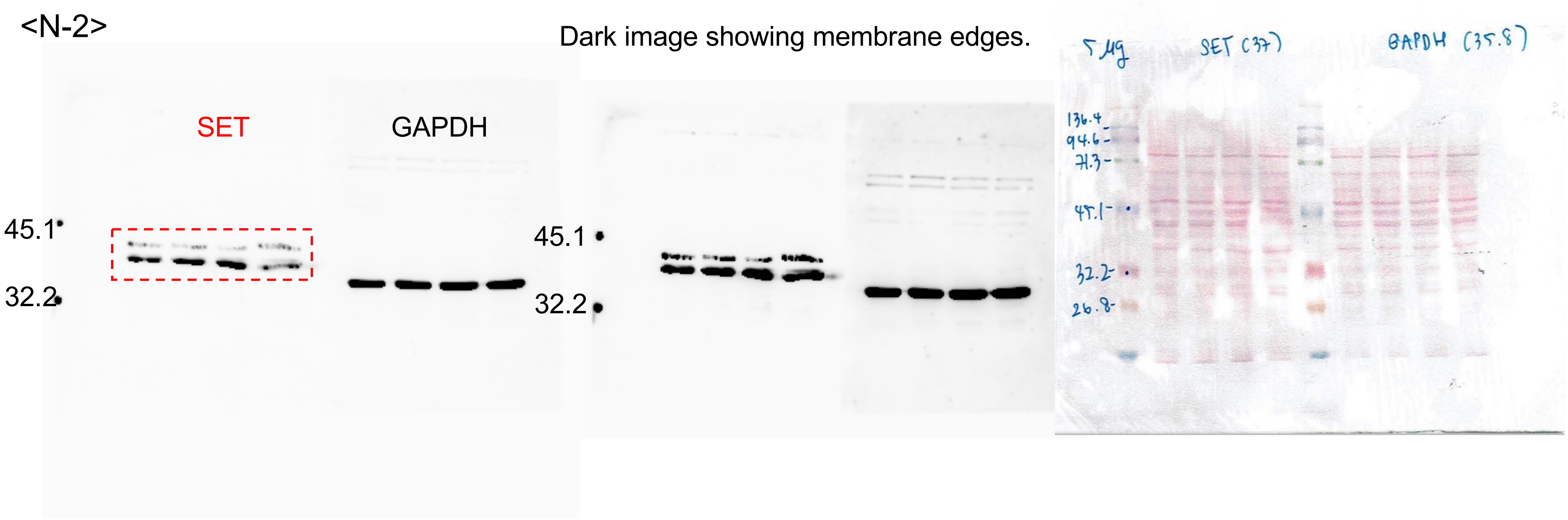

<N-3>

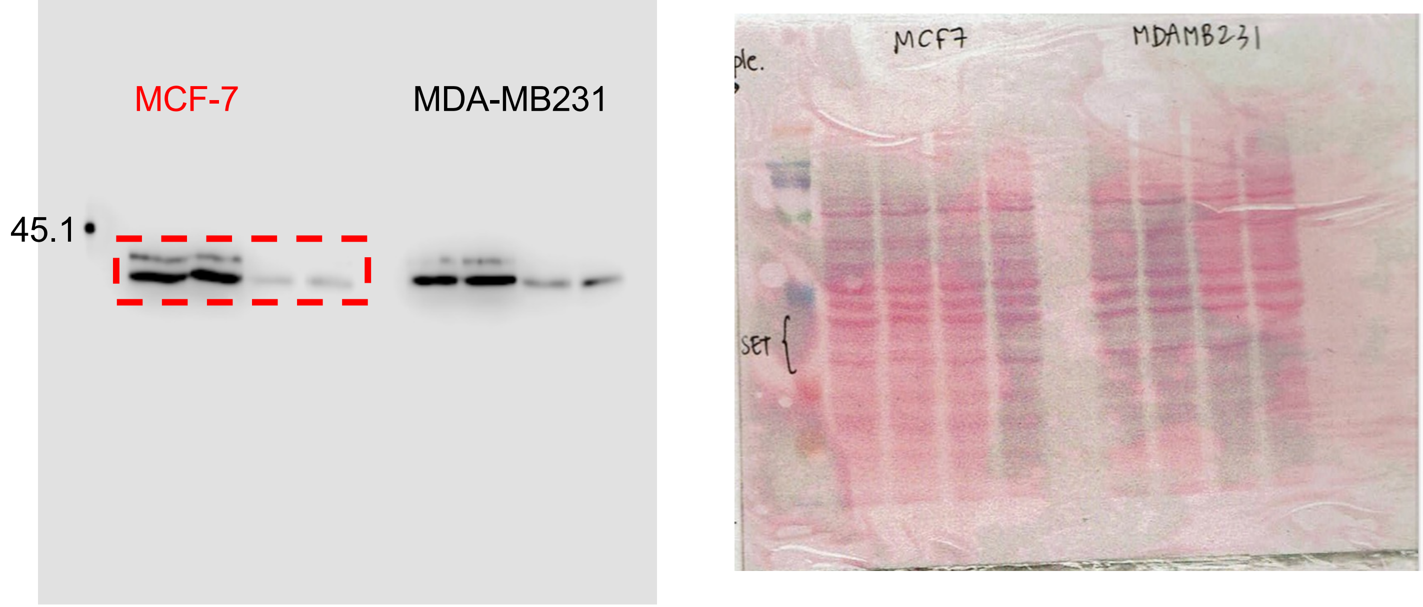

Figure S2- CIP2A of MCF-7 – Other replicates (N-2& N-3)

<N-2&3>

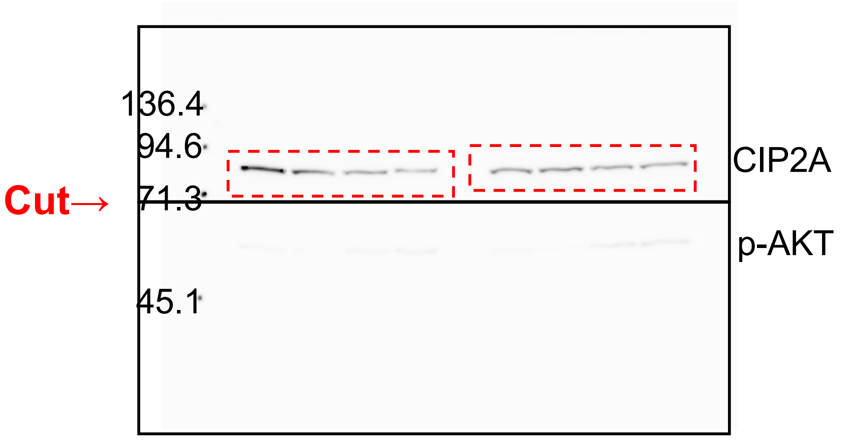

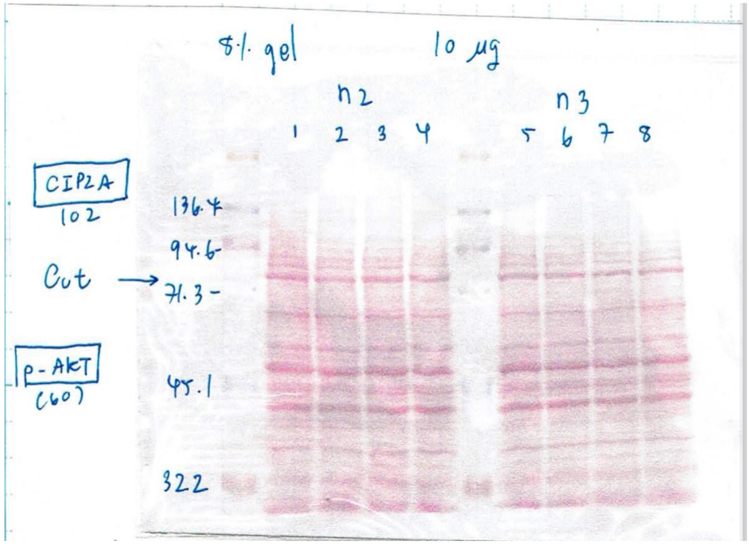

We cut the membrane prior to hybridization. All blots are indicated, but membrane edges are not clear.

Figure S2- GAPDH of MCF-7- Other replicates (N-2&N-3)

<N-2>

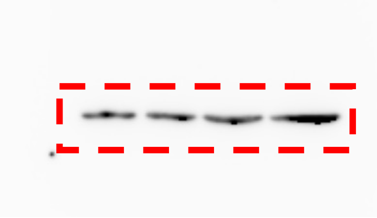

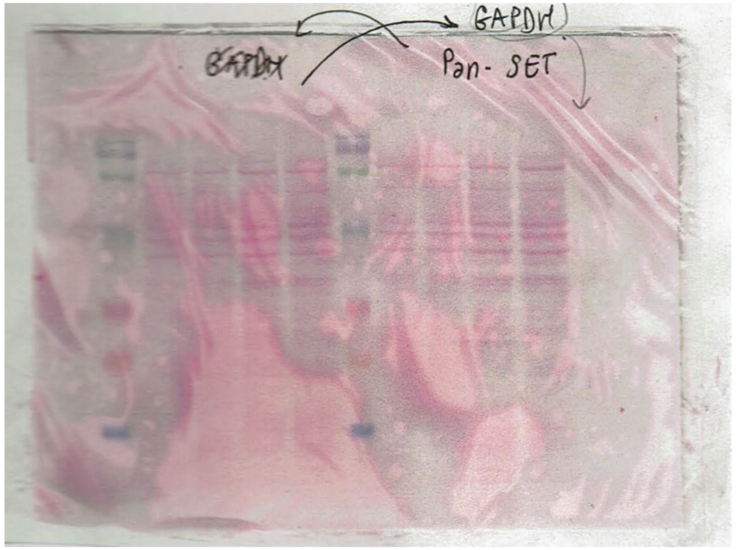

Multiple exposure images

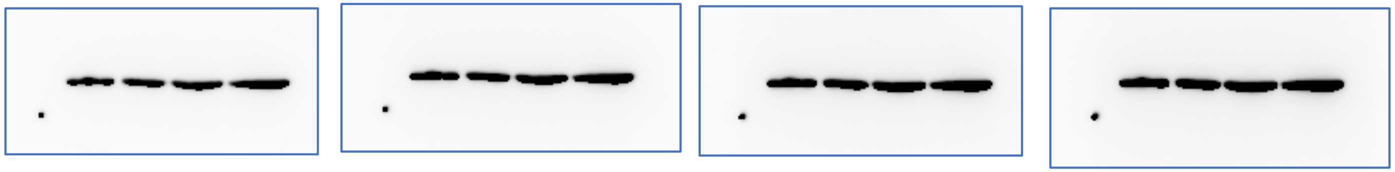

<N-3>

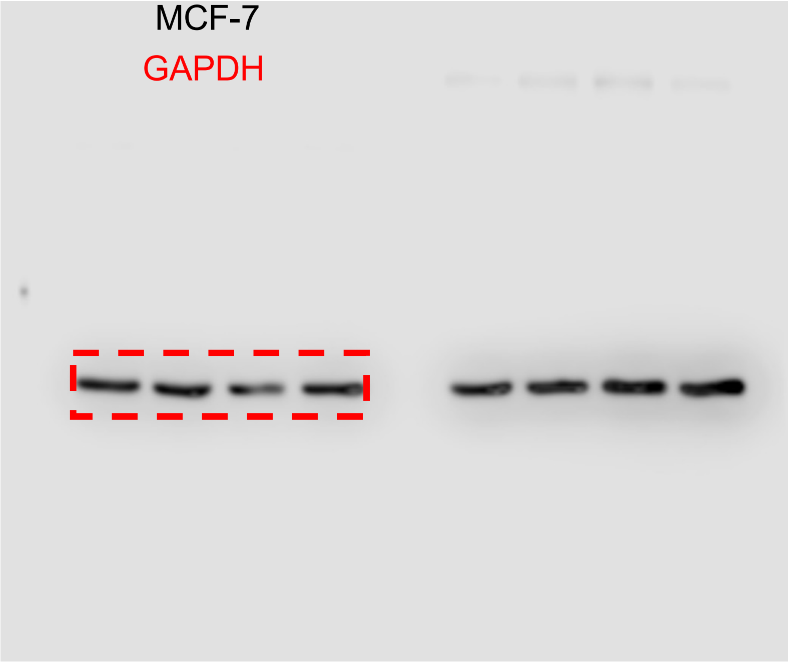

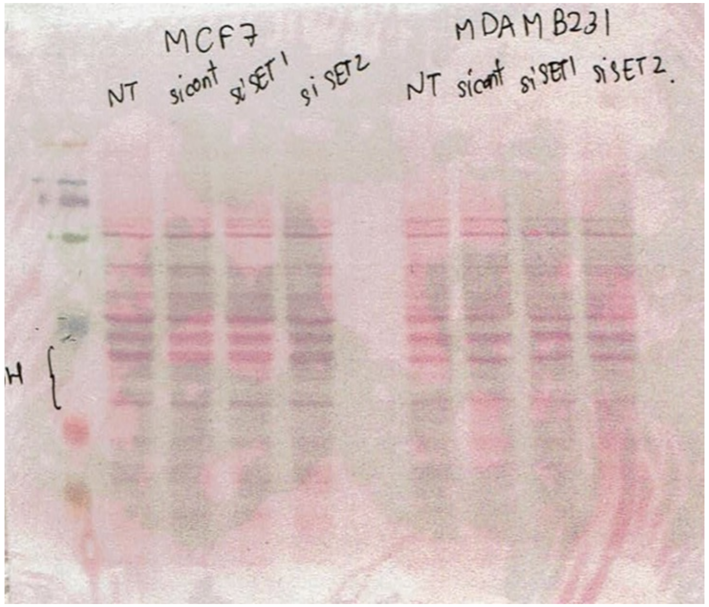

Figure S2- SET of MDA-MB-231 – Other replicates (N-2&N-3)

<N-2>

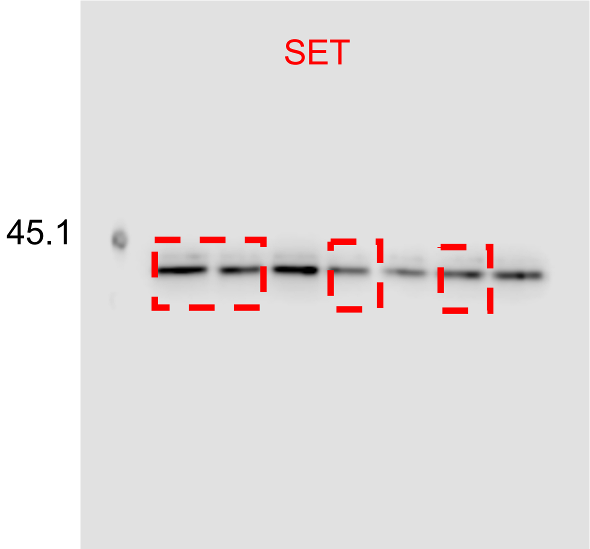

In this experiment, we examined 2 doses of each siCont, siSET-1 and siSET-2

Lane 1, non-treated
Lane 2, siCont
Lane 4, siSET-1
Lane 6, siSET-2

are correspond to Figure 2a.

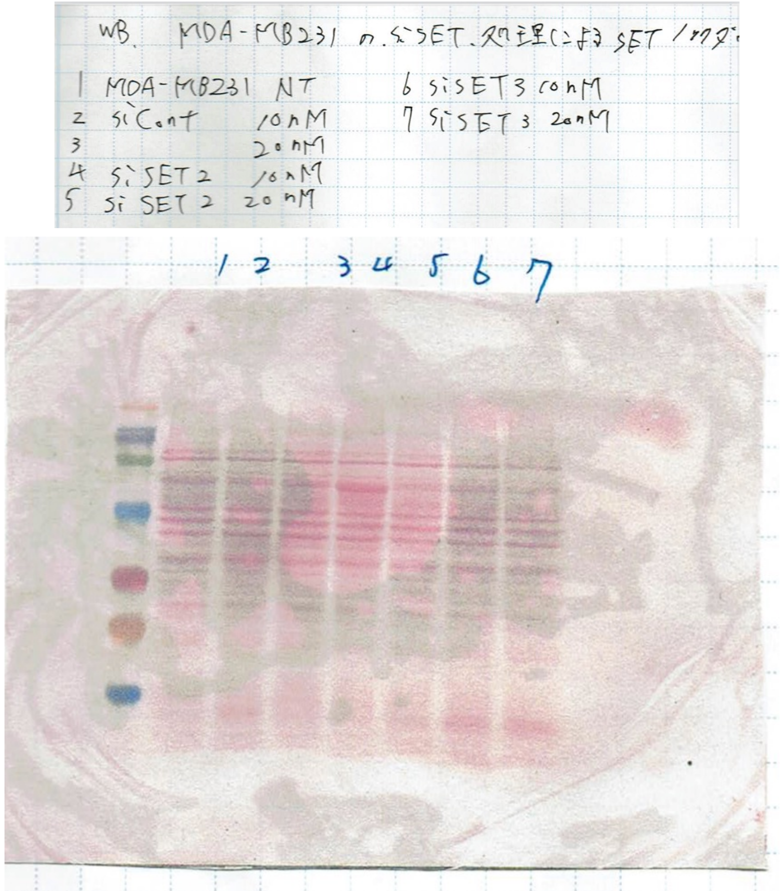

<N-3>

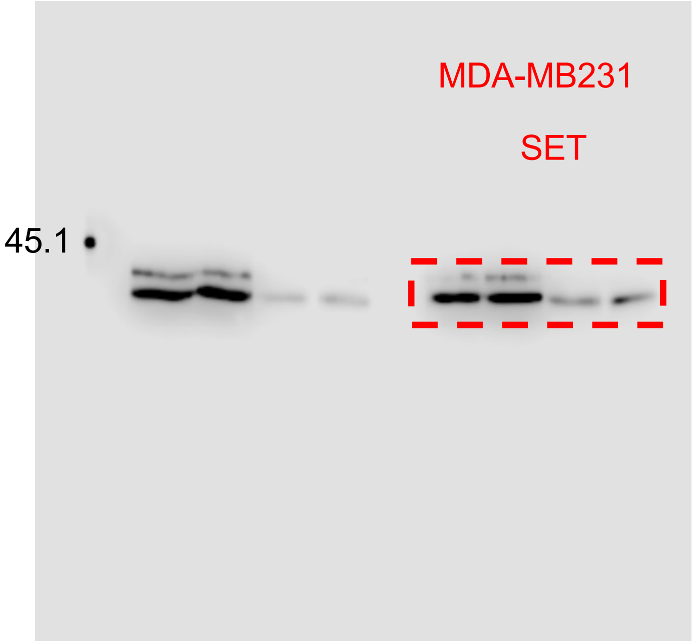

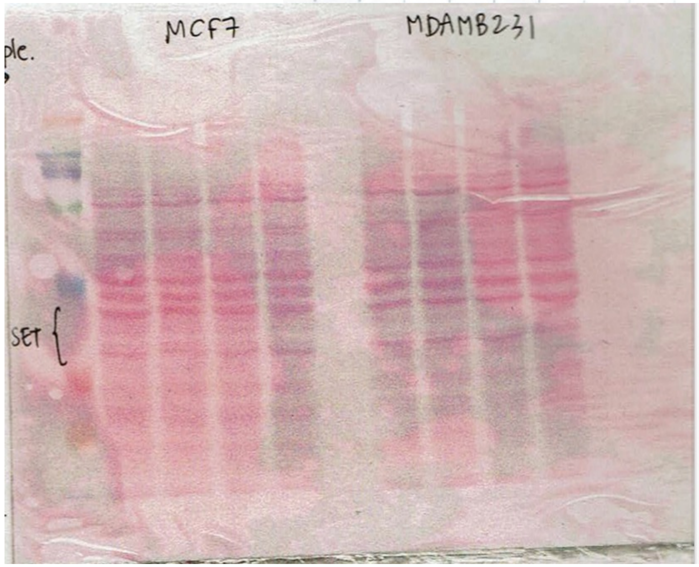

Figure S2- CIP2A of MDA-MB-231 – Other replicates (N-2 & N-3)

<N-2>

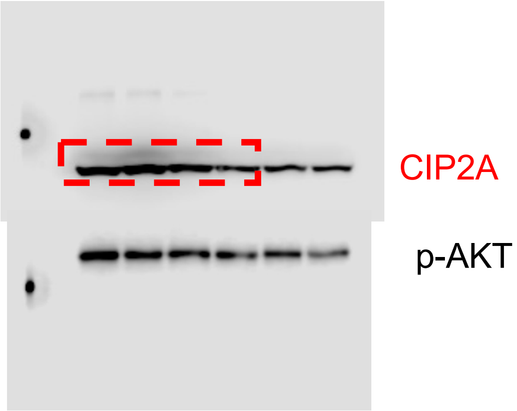

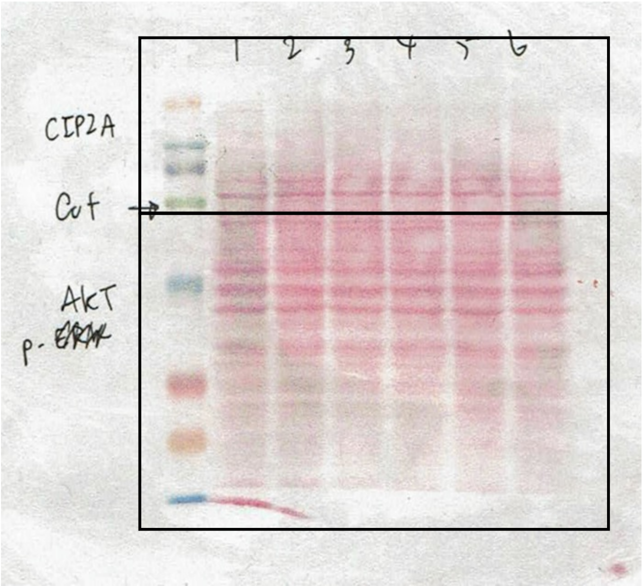

<N-3>

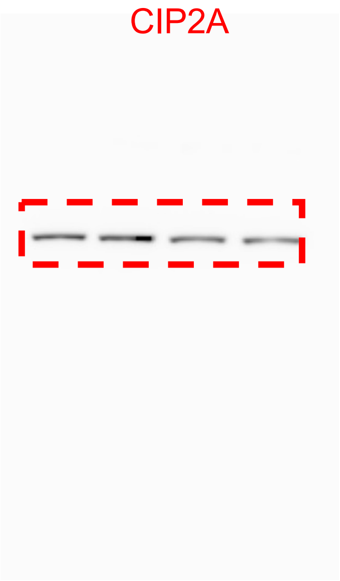

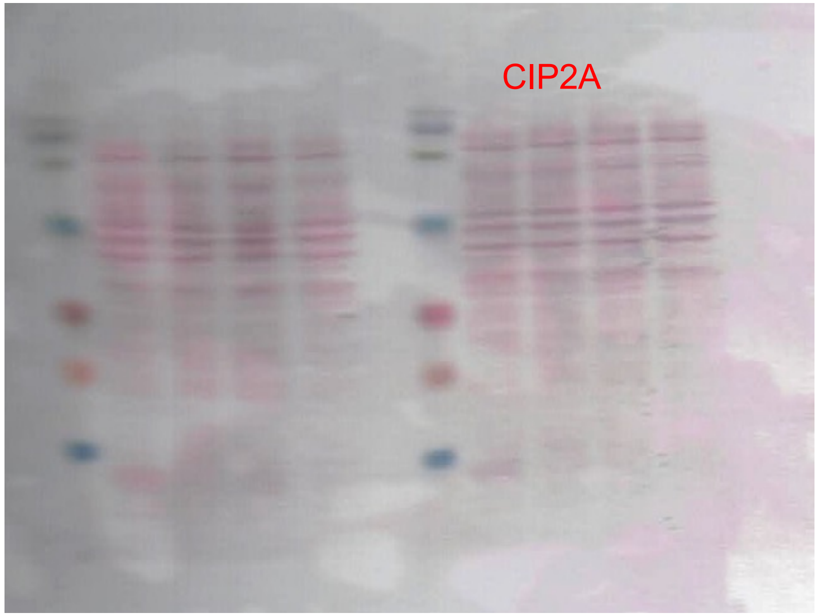

Figure S2- GAPDH of MDA-MB-231 – Other replicates (N-2&N-3)

<N-2>

GAPDH

Multiple exposure images

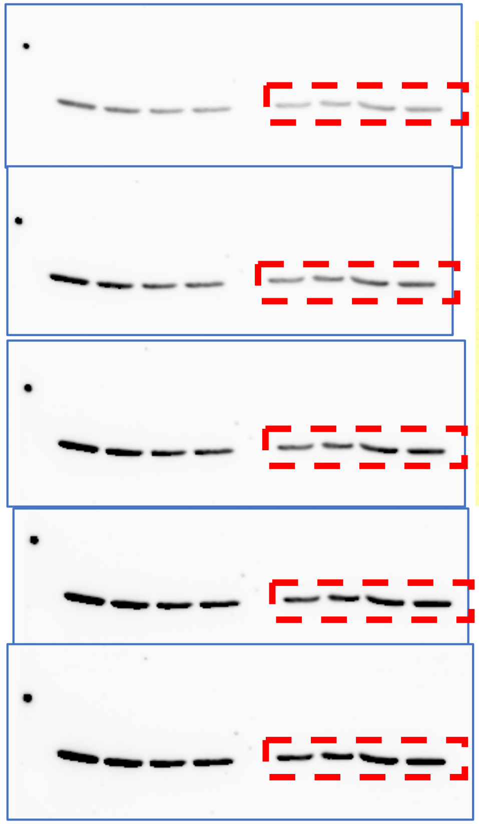

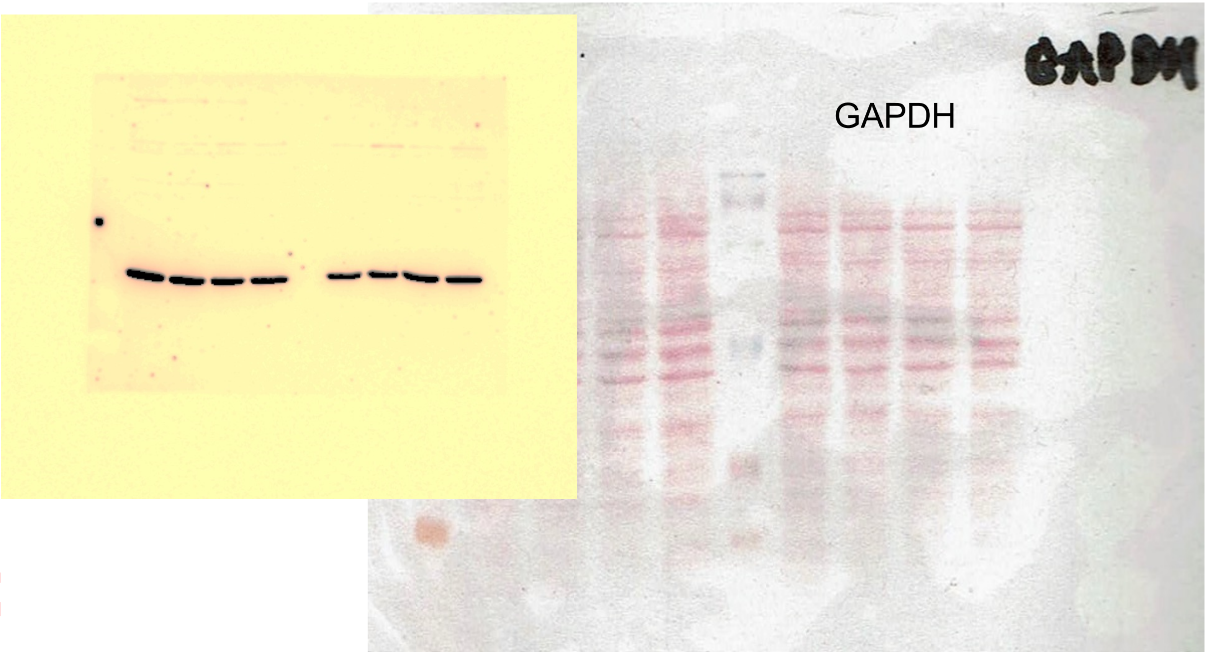

<N-3>

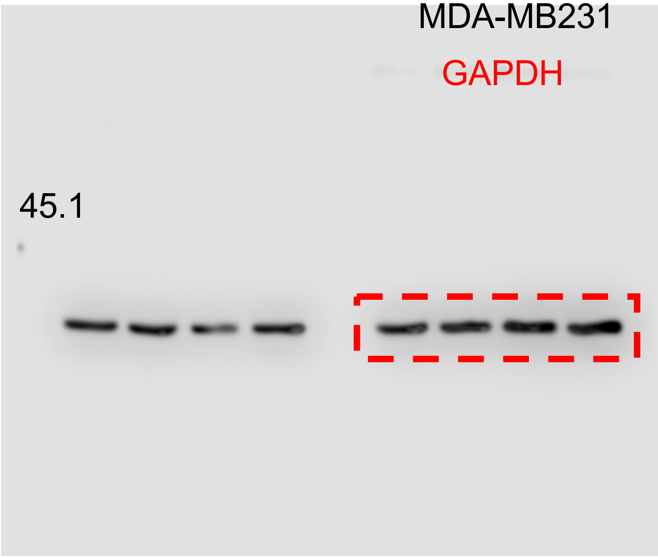

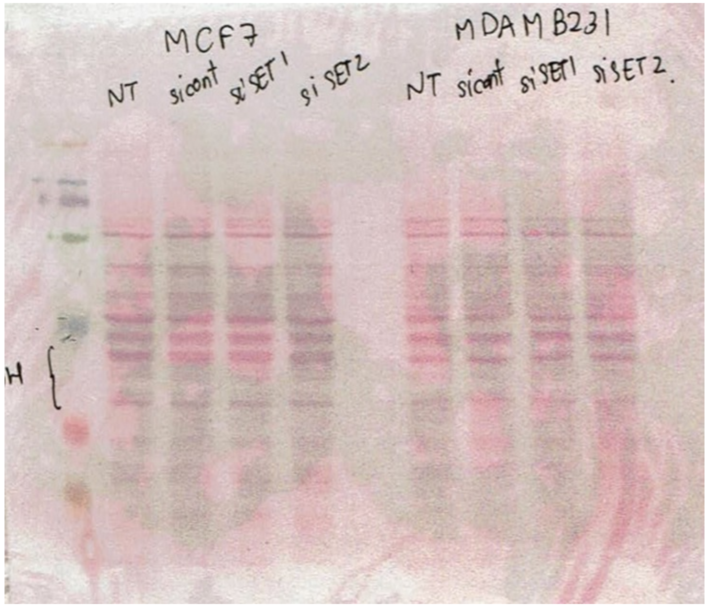

Supplement: Supplementary file 1 — Supplementary Information. [file 41598_2021_93620_MOESM1_ESM.pdf]
